# Dynamic Modeling and Experimental Validation of an Impact-Driven Piezoelectric Energy Harvester in Magnetic Field

**DOI:** 10.3390/s20216170

**Published:** 2020-10-29

**Authors:** Chung-De Chen, Yu-Hsuan Wu, Po-Wen Su

**Affiliations:** Department of Mechanical Engineering, National Cheng Kung University, Tainan City 701, Taiwan; n16064593@gs.ncku.edu.tw (Y.-H.W.); n16084462@gs.ncku.edu.tw (P.-W.S.)

**Keywords:** piezoelectric energy harvesting, vibration, frequency-up conversion

## Abstract

In this study, an impact-driven piezoelectric energy harvester (PEH) in magnetic field is presented. The PEH consists of a piezoelectric cantilever beam and plural magnets. At its initial status, the beam tip magnet is attracted by a second magnet. The second magnet is moved away by hand and then the beam tip magnet moves to a third magnet by the guidance of the magnetic fields. The impact occurs when the beam motion is stopped by the third magnet. The impact between magnets produces an impact energy and causes a transient beam vibration. The electric energy is generated by the piezoelectric effect. Based on the energy principle, a multi-DOF (multi-degree of freedom) mathematical model was developed to calculate the displacements, velocities, and voltage outputs of the PEH. A prototype of the PEH was fabricated. The voltages outputs of the beam were monitored by an oscilloscope. The maximum generated energy was about 0.4045 mJ for a single impact. A comparison between numerical and experimental results was presented in detail. It showed that the predictions based on the model agree with the experimental measurements. The PEH was connected to a diode bridge rectifier and a storage capacitor. The charges generated by the piezoelectric beam were stored in the capacitor by ten impacts. The experiments showed that the energy stored in the capacitor can light up the LED.

## 1. Introduction

The piezoelectric energy harvesters (PEHs) have received attention in the past two decays due to the high cost for battery replacements in the wireless sensor networks [1]. In the early development of PEHs, the resonators, such as cantilever beams, are used to harvest vibration energy from the environments [2,3,4]. The narrow bandwidth limits the application of the resonance-based energy harvesters. Many researchers have developed broad-band strategies to broaden the bandwidth of the resonance-based energy harvester, such as nonlinear oscillators, arrayed oscillators and multi-mode coupled oscillators [5].

In recent years, harvesting energy from human motions has become a hot topic because of the increasing demands of wearable devices [6]. The frequency of the human motion is extreme low, typically less than 5 Hz. For such low frequency, the energy conversion efficiency is also low for resonance-based energy harvesters. Although some broad-band strategies are adopted to enhance the efficiency, it is difficult to widen the bandwidth covering a frequency less than 5 Hz [7]. As an alternative, the concept of frequency-up conversion has been proven to be able to harvest energy at high efficiency from energy sources with extreme low frequencies. Umeda et al. presented a concept for energy harvesting based on the frequency up-conversion [8]. They investigate a ball free falling onto a piezoelectric beam to produce a transient vibration. Gu and Livemore presented a two-beam assembly [9]. The long beam vibrates in responding to the environment with a low frequency and hits the short piezoelectric beam with a high natural frequency. The electrical energy can be extracted from the transient vibration of the short beam with high efficiency. In recent years, some similar studies in energy harvesting based on frequency-up conversion with impact type can be also seen in [10,11,12,13].

In the above mentioned frequency up-conversion energy harvesters, the conversions from low frequency to high frequency through mechanical contact. Wickenheiser and Garcia presented a cantilever beam with magnet plucked by magnetic forces [14]. Although it avoids some problems such as surface wear and noises due to contact, the energy generated by the magnetic plucking is usually smaller than those based on mechanical contact. The frequency up-conversion driven by magnetic plucking forces was also adopted in some papers [15,16].

The frequency up-conversion concept has been proven having potential to harvest energy with extreme low frequency, and hence can be adopted to harvest energy from human motion. Pozzi and Zhu presented a device mounted at the knee to generate electrical energy from knee motion [17]. Their device is composed of a stator with plectra and a rotor with cantilever piezoelectric beams. As the knee moves, the stator and rotor have a relative rotational motion and the plectra pluck the beams. A refined device to harvest energy from knee motion was proposed by Kuang et al. [18]. The device is similar to that in [17] except that the beams were plucked by magnetic force. Their experiments showed that a maximum power of 4.5 mW can be harvested from walking. Wei et al. proposed an impact type energy harvester [19]. The presented this device mounted on a human leg to harvest energy from human walk. Their experiments showed that a power of 51 μW was generated for a walking speed of 5 km/h.

The magnetic forces are commonly used in the PEH designs. In the early development, the magnetic forces were assumed as simple forms such as inverse-square model [20,21,22]. These simple models are easy to implement. However, these forms valid only for some specific displacement range. For the case that the distance between two magnets are short or long, these simple models give inaccurate predictions. Some researchers assumed that the magnet as dipole without occupying a volume [23,24,25,26]. For the case of the large distance between two magnets, the model gives an accurate magnetic force. However, when one magnet approaches another, the assumption of the point-dipole become doubtful. Some 3D models have been proposed to calculate the interaction force between two magnets [27,28]. Comparing to the assumed form model and the point-dipole model, the 3D model requires more computation resources as it gives a more accurate prediction in magnetic force. In recent years, the 3D model has been introduced in developing the mathematical models of PEH [16,29,30,31].

In this paper, an impact-driven piezoelectric energy harvester (PEH) in the magnetic field is presented. A multi-DOF mathematical is developed to investigate the dynamic behaviors of the PEH. A 3-D magnetic force model is also introduced to calculate the magnetic forces between magnets. The voltage responses and energy harvested by the PEH can be calculated by the model. A prototype of the proposed design is fabricated to verify the numerical results of the model. Human motions, such as finger pressing, have been proven to trigger an PEH for driving a batteryless switch [26]. In this study, the PEH is driven by finger pressing and can be applicable to such applications.

This paper is organized as follows. In Section 1, the background of this study is presented. Some selected papers for piezoelectric energy harvesting are reviewed. The working principle of the PEH is mentioned in Section 2. In Section 3, the mathematical model for calculating the dynamic behaviors of the PEH are presented in detail. The fabrication and experimental setup of the PEH prototype are presented in Section 4. The discussions on the numerical and experimental results are presented in Section 5. The conclusions and findings of this study are summarized in Section 6.

## 2. Working Principle of the PEH

**Figure 1:**
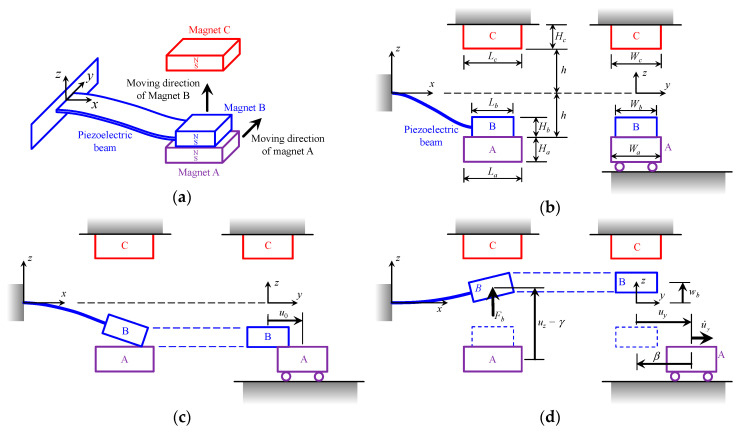
The conceptual drawing of the piezoelectric energy harvester (PEH). (**a**) The iso-view of the PEH at the initial status; (**b**) The side view and front view of the PEH at the initial status; (**c**) The PEH at the time that magnet B separates from magnet A; (**d**) The beam deflection before impact.

Figure 1a shows a conceptual drawing of the PEH, which includes a bimorph piezoelectric cantilever beam and three magnets. The magnetizations of the magnets are marked by N and S. The moving directions of the magnet A and the beam are also marked in Figure 1a. Figure 1b shows the side view and front view of the PEH. The magnet B is mounted at the beam’s tip. At its initial position, the beam magnet B is attracted on the moving magnet A. When magnet A moves along *y*-direction, the magnetic force acting on magnet B become smaller. At a critical position *u*_0_, the elastic force of the beam equals the magnetic force, and the beam magnet B is about to separate from magnet A, as shown in Figure 1c. The upward motion of the beam is driven by an upward force *F_b_*, which is the resultant of the magnetic forces from magnets A and C, and the elastic force of the beam bending. It should be noted that at the critical position *u*_0_, the beam magnetic force could produce a minor torque on the beam. It is assumed that the torque is small so that the induced torsion deformation can be neglected. During its upward-moving, the beam magnet B has a position *u_z_* and a velocity ${\dot{u}}_{z}$. Meanwhile, the magnet A has a position *u_y_* and a velocity ${\dot{u}}_{y}$, as illustrated in Figure 1d. Finally, the beam magnet B collides magnet C. After the impact, a transient vibration in the beam occurs and the electric energy is then extracted from the vibration due to the piezoelectric effect.

In the proposed PEH, the magnet B is moved by finger pressing. It means that the PEH is driven by human motion and could be applicable for driving a batteryless switch [26].

## 3. Dynamic Model of the PEH

**Figure 2:**
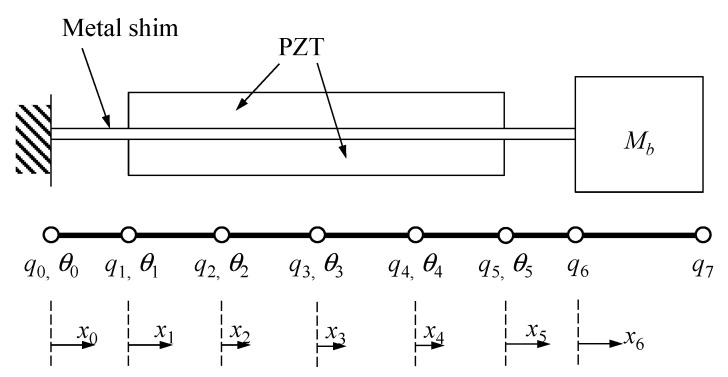
The beam model of the piezoelectric beam.

As shown in Figure 2, the piezoelectric beam is composed of a pair of PZT (lead zirconate titanate) and a middle metal shim. The beam is divided by 7 sub-beams. According to the motion sequence of the PEH, the analysis is divided into two phases. In the first phase, we consider the dynamic responses of the beam motion before the impact. In the second phase, the transient vibration after the impact is solved. In the rest of this section, the mathematical procedures of the two phases will be derived separately.

### 3.1. Analysis before Impact

The first phase of the model is to consider the beam motion before impact. In this phase, the acceleration is much smaller than that after impact so that we only consider the fundamental vibration mode of vibration, which is assumed to be the same as the static beam deflection subjected to a concentrated force at its end. Based on the Euler beam theory, the static beam deflection can be easily determined by the flexural formula. In a general form, the deflection can be written as

(1) $$w_{k}(x_{k},t)=w_{k}^{\left(1\right)}(x_{k},t)=\frac{w_{b}(t)}{U_{b}}W_{k}^{\left(1\right)}(x_{k}),\;k=0,1,\cdots 6$$

where the superscript (1) denotes the first phase, $w_{k}^{\left(1\right)}(x_{k},t)$ denotes the deflection of the *k*th sub-beam before impact, $W_{k}^{\left(1\right)}(x_{k})$ are the normalized deflection of the *k*th sub-beam under a unit force applied at the beam tip, and *U_b_* is the normalized deflection at the beam tip, i.e., $U_{b}=W_{6}^{\left(1\right)}(x_{6}=L_{6})$. The expressions for $W_{k}^{\left(1\right)}(x_{k})$ is given in the Appendix A. It is seen that *w_b_* actually denotes the deflection at the beam tip, i.e., $w_{b}(t)=w_{6}^{\left(1\right)}(x_{6}=L_{6})$.

By neglecting the small tilt angle *θ* of beam magnet B and the magnetic force along *x* and *y* directions, the force *F_a_*_-*b*_ along *z* direction acting on magnet B from magnet A can be written in the form [27]

(2) $$F_{a-b}=\frac{M_{a}M_{b}}{4\pi \mu_{0}}\left(\phi_{1}+\phi_{2}+\phi_{3}+\phi_{4}\right)$$

where *M_a_* and *M_b_* denote the magnetizations of the magnets A and B, respectively, $\mu_{0}\left(=4\pi \times 10^{-7}\;N/A^{2}\right)$ denotes the permeability of the air, and

(3) $$\phi_{1}=-\sum_{i=0}^{1}\sum_{j=0}^{1}\sum_{k=0}^{1}\sum_{l=0}^{1}\sum_{p=0}^{1}\sum_{q=0}^{1}\left[u_{ij}w_{pq}\ln\left(\sqrt{u_{ij}^{2}+v_{kl}^{2}+w_{pq}^{2}}-u_{ij}\right)\right]$$

(4) $$\phi_{2}=-\sum_{i=0}^{1}\sum_{j=0}^{1}\sum_{k=0}^{1}\sum_{l=0}^{1}\sum_{p=0}^{1}\sum_{q=0}^{1}\left[v_{kl}w_{pq}\ln\left(\sqrt{u_{ij}^{2}+v_{kl}^{2}+w_{pq}^{2}}-v_{kl}\right)\right]$$

(5) $$\phi_{3}=\sum_{i=0}^{1}\sum_{j=0}^{1}\sum_{k=0}^{1}\sum_{l=0}^{1}\sum_{p=0}^{1}\sum_{q=0}^{1}\left[u_{ij}v_{kl}tan^{-1}\left(\frac{u_{ij}v_{kl}}{w_{pq}\sqrt{u_{ij}^{2}+v_{kl}^{2}+w_{pq}^{2}}}\right)\right]$$

(6) $$\phi_{4}=-\sum_{i=0}^{1}\sum_{j=0}^{1}\sum_{k=0}^{1}\sum_{l=0}^{1}\sum_{p=0}^{1}\sum_{q=0}^{1}\left[w_{pq}\sqrt{u_{ij}^{2}+v_{kl}^{2}+w_{pq}^{2}}\right]$$

In Equations (3)–(6), the parameters *u_ij_*, *v_kl_*, and *w_pq_* are given by

(7) $$u_{ij}=\alpha +{\left(-1\right)}^{j}L_{a}-{\left(-1\right)}^{i}L_{b},\;i,\;j\;=\;0,\;1$$

(8) $$v_{kl}=\beta +{\left(-1\right)}^{l}W_{a}-{\left(-1\right)}^{k}W_{b},\;k,\;l\;=\;0,\;1$$

(9) $$w_{pq}=\gamma +{\left(-1\right)}^{q}H_{a}-{\left(-1\right)}^{p}H_{b},\;p,\;q\;=\;0,\;1$$

where (*L_a_*, *H_a_*, *W_a_*) and (*L_b_*, *H_b_*, *W_b_*) be the dimensions of the two magnets, (*α*, *β*, *γ*) is relative position vector from the center of magnet A to the center of magnet B. As shown in Figure 1d, it is seen that the components of the position vector are *α* = 0, *β* = −*u_y_* and *γ* = *u_z_* in this particular case.

Following a very similar procedure, *F_c_*_-*b*_, the *z*-component of magnetic force acting on the beam magnet B from magnet C, can be also calculated. The resultant force of the two magnetic forces is *F_b_* = *F_a_*_-*b*_ + *F_c_*_-*b*_. Initially, the magnetic force *F_b_* is greater than the elastic force of the beam and the magnet B is attracted by and at rest on the magnet A. When the magnet A begins to move upward along *y* direction, the magnetic force *F_b_* decreases. At a critical status *u_y_* = *u*_0_, the beam begins to move upward. During the upward motion, the kinetic energy $T^{\left(1\right)}$ elastic internal energy $V_{e}^{\left(1\right)}$ and electrostatic internal energy $V_{s}^{\left(1\right)}$ can be written in the following forms:

(10) $$T^{\left(1\right)}=\frac{1}{2}\sum_{k=0}^{6}\int_{0}^{L_{k}}{\left(\rho A\right)}_{k}{\left({\dot{w}}_{k}^{\left(1\right)}\right)}^{2}dx_{k}$$

(11) $$V_{e}^{\left(1\right)}=\frac{1}{2}\sum_{k=0}^{5}\int_{0}^{L_{k}}{\left(EI\right)}_{k}{\left(\frac{d^{2}w_{k}^{\left(1\right)}}{dx_{k}^{2}}\right)}^{2}dx_{k}-\frac{bd_{31}\left(t_{m}+t_{p}\right)V}{4s_{11}^{E}}\sum_{k=1}^{4}\int_{0}^{L_{k}}\frac{\partial^{2}w_{k}^{\left(1\right)}}{\partial x^{2}}dx_{k}$$

(12) $$V_{s}^{\left(1\right)}=\frac{bd_{31}\left(t_{m}t_{p}\right)V^{\left(1\right)}}{4s_{11}}\sum_{k=1}^{4}\int_{0}^{L_{k}}\frac{\partial^{2}w_{k}^{\left(1\right)}}{\partial x_{k}^{2}}dx_{k}+\left(\frac{\varepsilon_{33}s_{11}-d_{31}^{2}}{s_{11}}\right)\frac{b\left(L_{1}+L_{2}+L_{3}+L_{4}\right){\left(V^{\left(1\right)}\right)}^{2}}{4t_{p}}$$

where *V*^(1)^ denotes the output voltage of the PEH before impact, (*EI*)*_k_* is the bending rigidity of the *k*th sub-beam, *b* is the width of the beam, *d*_31_ is the piezoelectric constant, *s*_11_ is the compliance of the piezoelectric, *ε*_33_ is the dielectric constant of the piezoelectric, *t_m_* is the thickness of the metal shim, and *t_p_* is the thickness of the piezoelectric. The Lagrange equation for the 1-D motion can be written as

(13) $$\frac{d}{dt}\left(\frac{\partial L^{\left(1\right)}}{\partial {\dot{w}}_{b}}\right)-\frac{\partial L^{\left(1\right)}}{\partial w_{b}}=F_{b}$$

where the Lagragian *L*^(1)^ is defined by $L^{\left(1\right)}=T^{\left(1\right)}-V_{e}^{\left(1\right)}+V_{s}^{\left(1\right)}$. The current generated by the PEH is

(14) $$i^{\left(1\right)}=-\frac{bd_{31}\left(t_{m}+2t_{p}\right)}{2s_{11}}\sum_{k=1}^{4}\int_{0}^{L_{k}}\frac{\partial^{2}{\dot{w}}_{k}^{\left(1\right)}}{\partial x_{k}^{2}}dx_{k}+\frac{b\left(d_{31}^{2}-s_{11}\varepsilon_{33}\right)\left(L_{1}+L_{2}+L_{3}+L_{4}\right){\dot{V}}^{\left(1\right)}}{2t_{p}s_{11}}$$

If the PEH is connected to an external resistance *R*, the equation of circuit can be obtained by the Ohm’s law, i.e.,

(15) $$i^{\left(1\right)}=\frac{V^{\left(1\right)}}{R}$$

Substituting Equations (10)–(12) into Equation (13) and substituting Equation (14) into Equation (15), the equation of motion and equation of circuit before impact are

(16) $$m_{eq}{\ddot{w}}_{b}+k_{eq}w_{b}+\alpha V=F(u_{y},u_{z})$$

(17) $$\eta \alpha {\dot{w}}_{b}-C_{p}\dot{V}=\frac{V}{R}$$

where

(18) $$u_{z}(t)=w_{6}^{\left(s\right)}(x_{6}=L_{6}/2,t)+h+\frac{H_{a}}{2}$$

(19) $$\alpha =-\frac{bd_{31}\left(t_{m}t_{p}\right)}{4s_{11}U_{b}}\sum_{k=1}^{4}\int_{0}^{L_{k}}\frac{\partial^{2}W_{k}^{\left(s\right)}(x_{k})}{\partial x_{k}^{2}}dx_{k}$$

(20) $$\eta =\frac{2t_{m}+4t_{p}}{t_{m}t_{p}}$$

(21) $$C_{p}=\frac{b\left(L_{1}+L_{2}+L_{3}+L_{4}\right)}{2t_{p}}\left(\varepsilon_{33}-\frac{d_{31}^{2}}{s_{11}}\right)$$

Assume that the magnet A is moving at a constant velocity *v_a_*. Then the position of the magnet A is

(22) $$u_{y}=u_{0}+v_{a}\left(t-t_{0}\right)$$

where *t*_0_ is the time that the beam magnet B is about to separate from the magnet A. The initial condition at *t* = *t*_0_ is

(23) $$u_{y}=u_{0},\;w_{b}=-h-\frac{H_{a}}{2},\;{\dot{w}}_{b}=0$$

By solving Equations (16) and (17) with the initial conditions in Equation (23), the beam tip deflection *w_b_* and output voltage *V*^(1)^ before impact can be solved. By substituting the solved *w_b_* into Equation (1), the deflection curve of the piezoelectric beam before impact can be determined.

### 3.2. Analysis after Impact

At the instance of the impact, the deflection at the beam tip is $w_{b}=h-H_{b}/2$. By the use of Equation (1), the deflection of the beam at this instance is

(24) $$w_{k}^{\left(1\right)}(x_{k},t=0)=\frac{2h-H_{b}}{2U_{b}}W_{k}^{\left(1\right)}(x_{k}),\;k=0,1,\cdots 6$$

**Figure 3:**
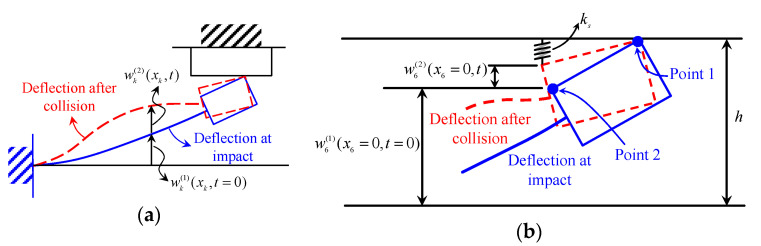
(**a**) Schematic of the beam deflection after impact; (**b**) The spring model of the contact.

Figure 3a shows the illustration of the deflection curve at the impact and after the impact. By introduce the dynamic displacement $w_{k}^{\left(2\right)}(x_{k},t)$ after the impact, the deflection of the beam $w_{k}^{\left(2\right)}$ after the impact can be written as

(25) $$w_{k}(x_{k},t)=w_{k}^{\left(2\right)}(x_{k},t)+w_{k}^{\left(1\right)}(x_{k},t=0),\;k=0,1,\cdots 6$$

Note that the second term in the right-hand-side of Equation (25) is independent of time so that the velocity is ${\dot{w}}_{k}(x_{k},t)={\dot{w}}_{k}^{\left(2\right)}(x_{k},t)$. The dynamic displacement $w_{k}^{\left(2\right)}(x_{k},t)$ can be written in linear combination of the interpolation functions $N_{k1}(x_{k})$ to $N_{k4}(x_{k})$, *k* = 0, 1, …, 6, i.e.,

(26) $$w_{k}^{\left(2\right)}(x_{k},t)=q_{k}^{\left(2\right)}(t)N_{k1}(x_{k})+\theta_{k}^{\left(2\right)}(t)N_{k2}(x_{k})+q_{k+1}^{\left(2\right)}(t)N_{k3}(x_{k})+\theta_{k+1}^{\left(2\right)}(t)N_{k4}(x_{k}),\;k=0,1,\cdots ,6$$

where $q_{k}^{\left(2\right)}(t)$ and $\theta_{k}^{\left(2\right)}(t)$ denote the dynamic displacement and dynamic rotation at the *k*th node for the dynamic term. The expressions for the interpolation functions are given in the Appendix A.

The magnet B may rebound after it impacts the magnet C. To estimate the rebound displacement, consider a simplified model that the magnet B approaches magnet C and collision occurs between them. In this simplified model, the beam is neglected. The rebound velocity of magnet B after impact is ${e{\dot{w}}_{b}|}_{w_{b}=h-H_{b}/2}$, where ${{\dot{w}}_{b}|}_{w_{b}=h-H_{b}/2}$ denotes the impact velocity of magnet B at the instance just before the impact and *e* is the coefficient of restitution during the impact. By knowing the mass of the magnet, the kinetic energy of the magnet B can be calculated according to the impact velocity. During the rebound, a negative work is done by the magnetic force. By using the work–kinetic energy principle, the rebound displacement can be estimated. It should be noted that the above simple model overestimates the rebound displacement because we neglect the effects from the beam, which has an upward momentum during the impact and reduce the downward rebound displacement. For the case of small rebound displacement, one can assume that the contact point (corner point 1 shown in Figure 3b) remains no separation after impact. The other corner point 2 shown in Figure 3b is allowed to have motion. However, the degree of freedom of this point is constrained by the magnetic force between two magnets. In this model, an equivalent spring *k_s_* between the magnets B and C is introduced to model this constraint, as shown in Figure 3b. For simplification without loss of generality, we set *t* = 0 at the instance of the impact. In the following, the analysis procedures of the beam after the impact will be derived in detail.

In the equivalent spring model, the spring force *F_s_*(*t*) is given by

(27) $$F_{s}(t)=k_{s}\delta (t)$$

where *k_s_* denotes the equivalent spring constant and

(28) $$\delta (t)=h-w_{6}^{\left(2\right)}(x_{6}=0,t)-w_{6}^{\left(1\right)}(x_{6}=0,t=0)$$

As an estimation, *k_s_* is written in the form

(29) $$k_{s}=\frac{dF}{d\delta }$$

To derive the equation of motion, consider the elastic internal energy $V_{e}^{\left(2\right)}$, electrostatic internal energy $V_{s}^{\left(2\right)}$ and kinetic energy. The equations of motion can be derived by Lagrange mechanics:

(30) $$\frac{d}{dt}\left(\frac{\partial L^{\left(2\right)}}{\partial {\dot{q}}_{k}^{\left(2\right)}}\right)-\frac{\partial L^{\left(2\right)}}{\partial q_{k}^{\left(2\right)}}=0,\;k=1,2,\cdots ,6$$

(31) $$\frac{d}{dt}\left(\frac{\partial L^{\left(2\right)}}{\partial {\dot{\theta }}_{k}^{\left(2\right)}}\right)-\frac{\partial L^{\left(2\right)}}{\partial \theta_{k}^{\left(2\right)}}=0,\;k=1,2,\cdots ,5$$

where $L^{\left(2\right)}=T^{\left(2\right)}-V_{e}^{\left(2\right)}-V_{e}^{\left(2\right)}$. The kinetic energy and electrostatic internal energy for the second phase have the same forms as those mentioned in Equations (10)–(12) by changing superscript (1) by (2). The elastic internal energy after impact is

(32) $$V_{e}^{\left(2\right)}=\frac{1}{2}\sum_{k=0}^{5}{\left(EI\right)}_{k}\int_{0}^{L_{k}}{\left(\frac{\partial^{2}w_{k}^{\left(2\right)}}{\partial x^{2}}\right)}^{2}dx_{k}-\frac{bd_{31}\left(t_{m}+t_{p}\right)V}{2s_{11}}\sum_{k=1}^{4}\int_{0}^{L_{k}}\frac{\partial^{2}w_{k}^{\left(2\right)}}{\partial x^{2}}dx_{k}+\frac{1}{2}k_{eq}{\left(h-q_{6}^{\left(2\right)}-q_{6}^{\left(1\right)}\right)}^{2}$$

Similarly, the circuit equation after impact can be derived in the same way. The equations of the motion and equation of circuit after impact can be written by

(33) $$M{\ddot{q}}^{\left(2\right)}+C{\dot{q}}^{\left(2\right)}+Kq^{\left(2\right)}+\alpha V^{\left(2\right)}=0$$

(34) $$\eta \alpha^{T}{\dot{q}}^{\left(2\right)}-C_{p}{\dot{V}}^{\left(2\right)}=\frac{V^{\left(2\right)}}{R}$$

where **M** is the mass matrix considering the masses of the beam and magnet, **C** is the damping matrix, **K** is the stiffness matrix considering both the elastic force of the beam and the equivalent spring, **α** is the electromechanical coupling matrix containing the piezoelectric constant, *C_p_* is the capacitor of the PZT, *V* is the output voltage, *R* is the resistance, and

(35) $$q^{\left(2\right)}={\left[\begin{array}{cccccc} q_{1}^{\left(2\right)} & \theta_{1}^{\left(2\right)} & \cdots & q_{5}^{\left(2\right)} & \theta_{5}^{\left(2\right)} & q_{6}^{\left(2\right)} \end{array}\right]}^{T}$$

The expressions for the matrices **M**, **K** and **α** are given in the Appendix A. The proportional damping model is used in the present analysis, i.e.,

(36) $$C=\gamma_{1}M+\gamma_{2}K$$

where *γ*_1_ and *γ*_2_ are constants and can be determined by solving the following two equations:

(37) $$\frac{\gamma_{1}}{\omega_{1}}+\omega_{1}\gamma_{2}=2\zeta_{1}$$

(38) $$\frac{\gamma_{1}}{\omega_{2}}+\omega_{2}\gamma_{2}=2\zeta_{2}$$

where *ω_k_*, *ζ_k_* (*k* = 1, 2) are the natural frequency and damping ratio, respectively, of the first two vibration modes.

The boundary conditions for the dynamic displacement at *x*_0_ = 0 and *x*_6_ = *L*_6_ after the impact are

(39) $$q_{0}^{\left(2\right)}=0,\;\theta_{0}^{\left(2\right)}=0,\;\theta_{7}^{\left(2\right)}=0$$

At the instance that the impact occurs (*t* = 0), the initial conditions for the second phase are

(40) $$\begin{array}{c} q_{k}^{\left(2\right)}(t=0)=0,\;\theta_{k}^{\left(2\right)}(t=0)=0,\;{\dot{q}}_{k}^{\left(2\right)}(t=0)=\frac{1}{U_{b}}W_{k}^{\left(1\right)}\left({{\dot{w}}_{b}|}_{w_{b}=h-\frac{H_{b}}{2}}\right),\\ {\dot{\theta }}_{k}^{\left(2\right)}(t=0)=\frac{1}{U_{b}}\frac{dW_{k}^{\left(2\right)}}{dx_{k}}\left({{\dot{w}}_{b}|}_{w_{b}=h-\frac{H_{b}}{2}}\right),\;V^{\left(2\right)}(t=0)={V^{\left(1\right)}|}_{w_{b}=h-\frac{H_{b}}{2}} \end{array}$$

where ${{\dot{w}}_{b}|}_{w_{b}=h-\frac{H_{b}}{2}}$ denotes the velocity of the beam tip at the time just before the impact and can be determined from the solution of Equation (13) mentioned in phase 1.

By solving Equations (33) and (34) in conjunction with the boundary conditions Equation (39) and initial conditions Equation (40), the transient responses of displacements and voltage of the beam can be obtained.

The energy *E*_PEH_ harvested by the PEH can be calculated by

(41) $$E_{\mathrm{PEH}}=\int_{t_{0}}^{0}\frac{V^{2}}{R}dt+\int_{0}^{\Delta t}\frac{V^{2}}{R}dt$$

where Δ*t* is the time interval for calculating the energy.

## 4. Fabrication of Prototype and Experimental Setup

**Figure 4:**
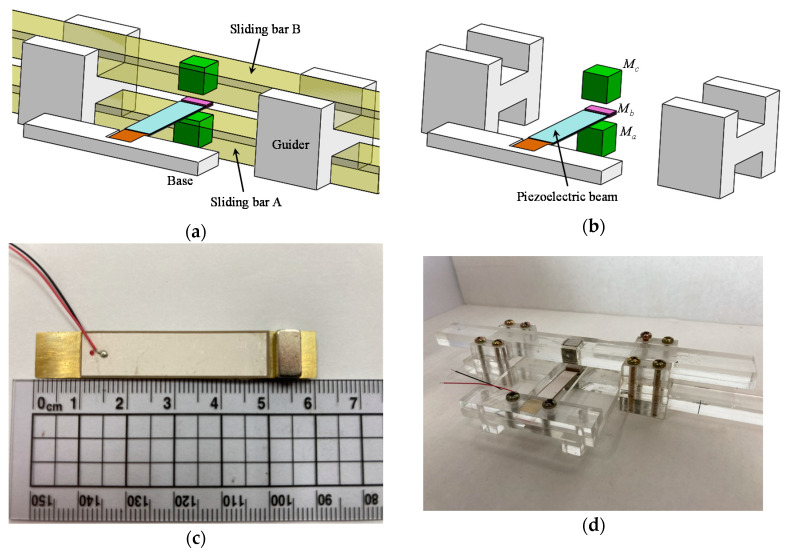
(**a**) The design of the PEH; (**b**) The design of the PEH with invisible slider bars; (**c**) Piezoelectric beam; (**d**) Prototype of the PEH.

Figure 4a illustrates the PEH prototype. The piezoelectric beam is clamped on a base. The magnets A and C are respectively mounted on two sliding bars, which can slide under the guidance of a pair of guiders. In the initial status, the beam magnet B is attracted by magnet A and is at rest. The upper sliding bar is placed at a position that the magnet C is at the position aligned to magnets A and B. By pushing the sliding bar to move right, the magnet A separates magnet B, and the beam moves upward and finally stopped by the magnet C. Then an impact occurs. In Figure 4b, the two bars are hidden for a better view for piezoelectric beam and magnets. The base, guiders and bars are made by acrylic to avoid interfering the magnetic fields.

The fabrication and assembly of the piezoelectric beam were provided by Eleceram Technology Co., Taiwan, as shown in Figure 4c. The geometric parameters of the beam and magnets are listed in Table 1. Two identical NdFeB magnets with a size of 10×5×0.8 mm^3^ were adhered at the beam tip to form the magnet B, which has an equivalent size of 10×5×1.6 mm^3^. Another two identical NdFeB magnets serve as magnets A and C, each of which has a size of 10×10×10 mm^3^. The geometric parameters of the magnets are also summarized in Table 1. Figure 4d shows the assembled prototype.

**Figure 5:**
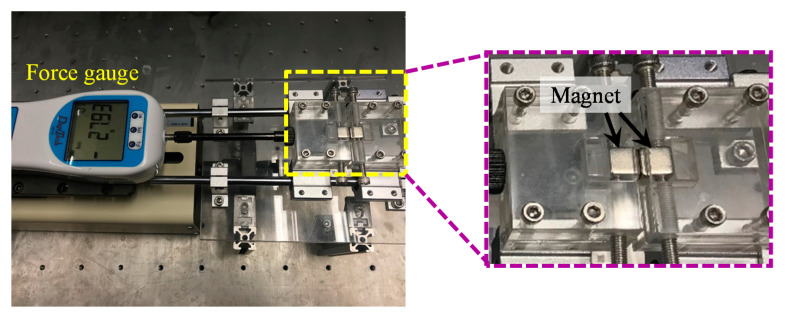
The experimental setup for the magnetic force measurement.

Figure 5 shows the setup for measuring the magnetic forces from one magnet to another for various distances between two magnets. In the experiment, a magnet was fixed on the fixture. A second magnet was placed on a jig that can freely slide along a guider. A force gauge (DigiTech DTG-10) withstood the second magnet at a gap between the two magnets and measured the magnetic force. Three magnet pairs A-A, B-B and A-B were considered in the magnetic force measurements for various gaps between the two magnets.

For the magnet pair A-A experiment, the magnetic force $F_{a-a,k}^{\left(\exp\right)}$ is measured for *k*th gap. Based on the 3-D magnetic force model described in Equation (2), the magnetic force $F_{a-a,k}^{\left(\mathrm{model}\right)}$ of the magnet pair A-A can be written in the form:

(42) $$F_{a-a,k}=\frac{M_{a}^{2}}{4\pi \mu_{0}}\left(\phi_{1,k}+\phi_{2,k}+\phi_{3,k}+\phi_{4,k}\right)$$

where the subscript *k* denotes the parameters calculated according to the *k*th gap. According to the least square method, the magnetic magnetization *M_a_* of the magnet A can be determined solving the equation:

(43) $$\frac{d}{dQ_{a-a}}\sum_{k=1}^{N}{\left(F_{a-a,k}^{\left(\exp\right)}-F_{a-a,k}\right)}^{2}=0$$

where $Q_{a-a}=M_{a}^{2}$ and *N* denotes the number of gaps for the measurement. Following a similar procedure, the magnetization *M_b_* can be also determined from the magnet pair B-B experiment.

**Figure 6:**
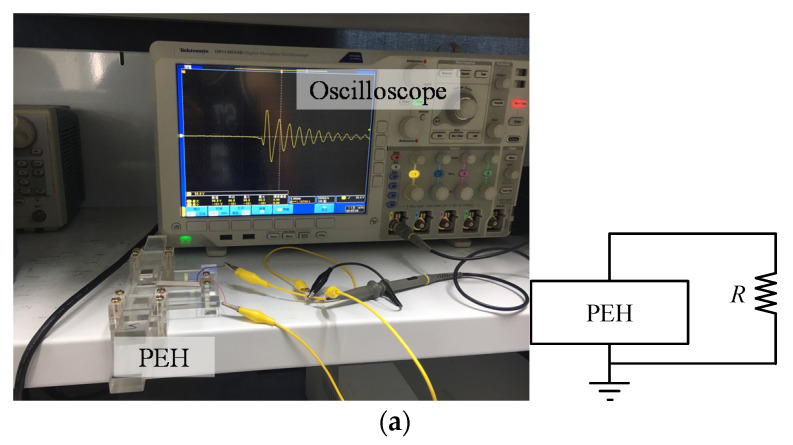

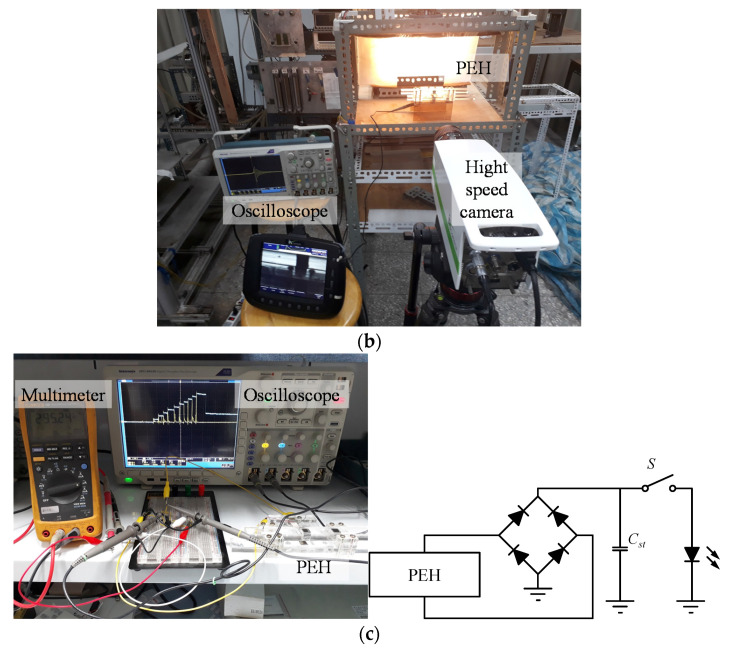
The experimental setup of the PEH: (**a**) voltage measurement of the PEH coupled with a resistance; (**b**) motion measurement of the PEH by the highspeed camera; (**c**) voltage measurement of the PEH coupled with a diode-bridge, capacitor, and LED.

To investigate the energy generated by the PEH, an oscilloscope (Tektronix DPO 4054B) was used to monitor the output voltage of the resistor connected to the PEH, as shown in Figure 6a. Because the motion of the sliding bar is moved by hand, the velocities of the magnet A change for different tests. The motion of the sliding bar (or the magnet A) was monitored by a high-speed camera (OLYMPUS i-SPEED 3) with a frame rate of 5000 Hz, as shown in Figure 6b. By analyzing the photos frame by frame, the positions of the sliding bar and the beam tip can be determined. The velocities of the beam magnet B can be calculated accordingly.

Figure 6c shows experimental setup for light LED. Four diodes (1N4004) were connected to form a bridge. The AC voltages were rectified to DC signals and the electric charges were stored in a capacitor *C_st_* of 10 μF. An LED was connected to the capacitor in parallel. In the experiments, the first impact was driven by magnet A and the collision between the magnets B and C. Put the magnet A back to the initial position and move the magnet C. Then the beam moved to magnet A and the second impact occurred. The process was repeated ten times and produced ten impacts. For each impact, the capacitor was charged and the voltage across the capacitor was boosted. After ten impacts, the switch S was switched to connect the LED loop. The capacitor discharged and a current flowed through and lighted up the LED. The oscilloscope was used to monitor the voltage across the capacitor during the charging and discharging in order to evaluate the energy stored in the capacitor. A multimeter (Fluke 189) was used to capture the peak current during the discharging in order to evaluate the maximum instantaneous power for lighting the LED.

## 5. Results and Discussions

In this section, the experimental and numerical results are presented to demonstrate the performance of the PEH. The material properties used in the dynamic model are listed in Table 2.

### 5.1. Measurements of Magnetic Forces

**Figure 7:**
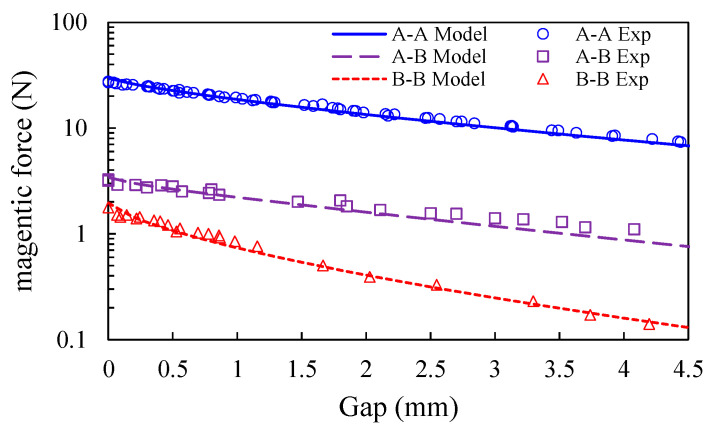
The comparisons of magnetic forces by measurements and model for magnet pairs A-A, B-B, and A-B for various gaps.

The variation of the magnetic forces for various gaps are shown in Figure 7. Note that the magnetizations *M_a_*, *M_b_*, and *M_c_* for the magnets A, B, and C are respectively unknown. By using the least square method described in Equation (43), the magnetizations *M_a_* and *M_b_* are determined to be 0.9537 T and 0.5147 T, respectively. In Figure 7, it is seen that the regression curves for magnet pairs A-A and B-B agree well with the measurement data. These two magnetizations are used to calculate the magnetic forces for the magnet pair A-B. In Figure 7, it is seen that the computation results for magnetic force between magnets A and B are validated by experiments. It indicates that Equation (2) can used to predict the magnetic force at high accuracy.

### 5.2. Effects of the Velocity of the Magnet A

**Figure 8:**
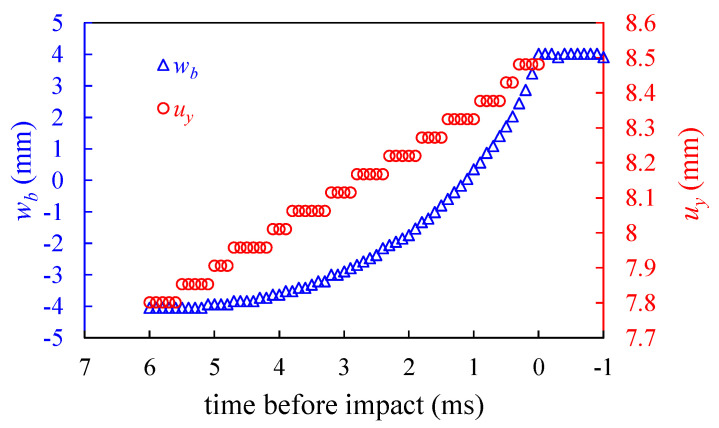
The measured deflection *w_b_* of the beam tip and the position *u_y_* of the magnet A before impact for the first test.

The PEH was driven by finger pressing. The velocity *v_a_* of the magnet A varies for different tests. To investigate the effects of the velocity *v_a_* on the motion of the beam and output voltage, five tests to trigger the PEH have been tested. Figure 8 shows the measured beam tip deflections *w_b_* for the first test. The deflection results show that the beam tip accelerated during its upward motion before impact. The measured positions *u_y_* of the magnet A for the first test are also showed in Figure 8. It is seen that the position *u_y_* of the magnet A exhibits a linear trend over time and the constant velocity assumption described in Equation (22) can be acceptable.

Table 3 lists the measured relative position *u*_0_ between magnets A and B at the instance that the beam tip begins to move upward, and the measured velocities *v_a_* of the magnet A for the five tests. The variation of relation position *u*_0_ for different tests is small. Meanwhile, the velocity *v_a_* varies from different tests because the motion of the magnet A was driven by hand. Among the five tests, the maximum, minimum and average values of *v_a_* are 0.1337 m/s, 0.07763 m/s, and 0.09731 m/s, respectively. The measured impact velocity, which is defined as the beam tip velocity ${{\dot{w}}_{b}|}_{w_{b}=h-\frac{H_{b}}{2}}$ at the time just before impact, for the five tests are also listed in Table 3. It is observed that the relation between impact velocity and the moving magnet velocity *v_a_* is insignificant. The average impact velocity for the five tests is 5.106 m/s. Assume that the coefficient of restitution during the impact is *e* = 0.5, then the rebound velocity of the magnet B is 2.553 m/s. By knowing the mass of 0.584 g for the magnet B, the kinetic energy after impact is 1.955 mJ. According to the magnetic force measurements, the magnetic attraction force is approximately 3 N when the magnet B is close to magnet C. By using the energy balance, the rebound displacement is 0.65 mm, which is quite small by comparing the whole moving distance. As mentioned in Section 3.2, the calculation of the rebound displacement is overestimated. It can be concluded that the small rebound assumption is acceptable. In Figure 8, the measurement data also show the beam tip deflection after impact. It is observed that the beam tip becomes stationary after impact, i.e., no rebound can be observed. Therefore, the model shown in Figure 3b can be acceptable.

**Figure 9:**
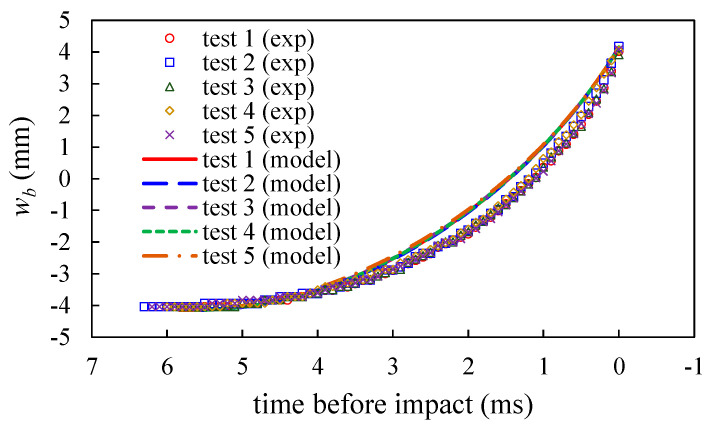
The comparisons of the deflection *w_b_* of the beam tip before impact obtained by experiments and model.

Figure 9 shows the time history of the measured beam tip deflection for the five tests. Each test reveals almost identical beam tip deflection curve. It indicates that the beam tip motion is independent of the driving velocity *v_a_*. The model calculation results agree well with the measured data, indicating that the model for the first phase can predict the motion of the beam tip at high accuracy.

**Figure 10:**
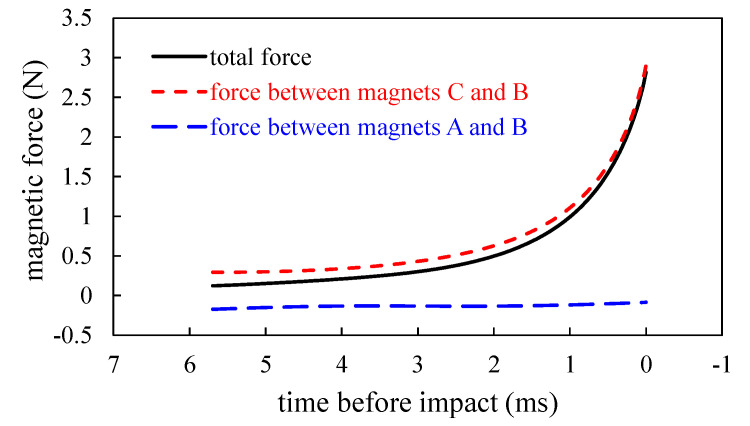
The computed magnetic forces by 3-D magnetic force model before impact.

Figure 10 shows the time history of magnetic forces applied on magnet B from magnets A and C during the phase before impact. As expected, the magnetic force from magnet C increases by time, while the magnetic force from magnet A is relatively small, especially for the time approaching impact. At the time of 1 ms before impact, the magnetic force from magnet C dominates the beam tip motion. The small force magnetic force from magnet A can be used to explain the insignificant contribution of *v_a_*.

**Figure 11:**
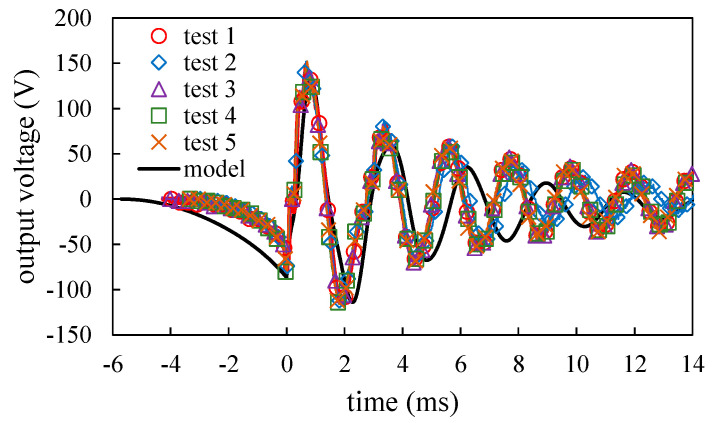
The comparisons of the open-circuit voltage responses of the PEH obtained by experiments and the dynamic model.

Figure 11 shows the open-circuit voltage for the five tests. The transient responses of the voltages before and after impact keep the same for various *v_a_*. The voltage responses computed from the model are also shown in Figure 11. According to the voltage responses after impact shown in Figure 11, the motion mainly exhibits the fundamental mode although some minor higher order vibration modes are observed. The domination of the fundamental mode can be also seen in model calculations. It is seen that the model agrees with the measurements. By picking the peak and valley points of the transient voltage response, the natural frequency of the vibration can be determined. According to this method, the natural frequencies for the five tests in Figure 11 are 407.9, 403.3, 408.9, 413.6, and 410.9 Hz. The average natural frequency for the five data is 408.9 Hz. The same method can be also applied in the time response obtained by the model and the result is 373.0 Hz.

### 5.3. Energy Measurements of the PEH

**Figure 12:**
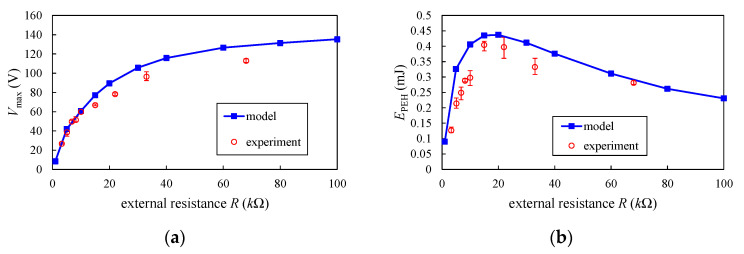
(**a**) The maximum output voltage of the PEH for various external resistance *R*; (**b**) The energy harvested by the PEH for various external resistance *R*.

Figure 12 shows the measured maximum *V*_max_ and energy *E*_PEH_ harvested by the PEH for various external resistances. For small external resistance, both *V*_max_ and *E*_PEH_ are small. The energy increases with the increase of *R* when *R* is less than 20 *k*Ω. A maximum energy of 0.4045 mJ occurs at the optimum resistance *R*_opt_ = 15 *k*Ω. For a large *R*, *V*_max_ reaches a stable value and the average power becomes small. In Figure 12, the measured voltage and energy are also compared with the results computed by the model. The comparisons show that the results of the model agree well with the experimental data.

**Figure 13:**
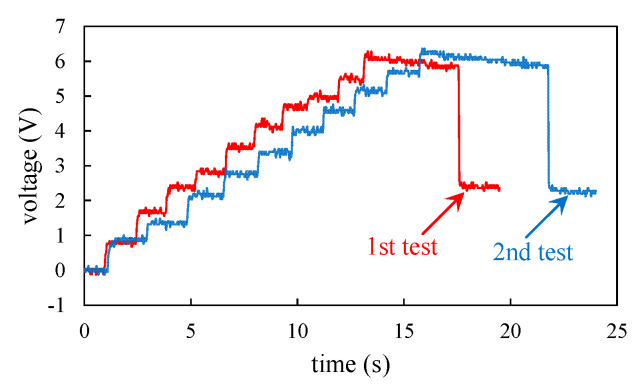
The measured voltage of the capacitor in the lighting up LED experiment.

For the lighting up LED experiment, two tests were conducted and the voltages across the capacitor, as shown in Figure 6c, are plotted in Figure 13. For each test, the voltage was boosted to 6.2 V after ten impacts and the energy stored in the capacitor was 19.22 μJ. When the circuit was switched, the capacitor discharged then a current flowed through and lighted up the LED. The voltage drops during the discharging for the two tests were 3.44 V and 3.52 V. The maximum currents during the discharging for the two tests were 450 μA and 476 μA. The maximum instantaneous powers during the discharging for the two tests were 0.774 mW and 0.838 mW. The experiments showed that the energy stored in the capacitor can light up the LED.

## 6. Conclusions

In this study, the impact-driven PEH in the magnetic field is presented. The multi-DOF mathematical model was developed for solving the dynamic and vibration behaviors of the piezoelectric beam under the effects of magnetic fields. The prototype was also fabricated and the performance of the PEH was measured in detail. The conclusions of this study are summarized as follows:

(1) The 3-D magnetic force model were introduced to calculate the magnetic force between magnets. The magnetic force experimental setup was developed and the measured forces for various gaps between two magnets agree with the model.
(2) Based on the multi-DOF mathematical model, the deflections and voltages of the piezoelectric beam were investigated in detail. The model is divided by two phases. In the first phase, the motion of the piezoelectric beam is governed by the restoring force of the beam and the magnetic forces due to magnets. The second phase begins at the time of impact. The ending conditions of the first phase are imposed as the initial conditions in the analysis of the second phase. In the second phase, the transient vibration responses can be solved.
(3) To produce the impact, the magnet A was moved by hand and the consequent motions were triggered. The experimental results showed that the velocity *v_a_* of magnet A varies from different tests. However, it was found that the variations of *v_a_* have nearly no contribution on the beam motion in the first phase and the voltage responses in the second phase. This phenomenon was also observed in the model simulation.
(4) The voltage and energy outputs were measured for various external resistance *R*. The experiments showed that the voltage outputs increases with the increase of *R*. The energy output was observed to be low for both small and large *R*. The maximum energy output was found to be 0.4045 mJ at the optimum resistance *R*_opt_ = 15 *k*Ω. The voltage and energy outputs computed by the model for various resistances agree well with the measurements.
(5) In the lighting LED experiment, the voltage was charged and an energy of 19.22 μJ was stored in the capacitor by ten impacts. The experiments showed that the energy stored in the capacitor can light up the LED.
(6) The permanent magnets are brittle and easy to be damaged after impact. In the experiments, however, no cracks or damages have been observed in the magnets. It suggests that the impact velocity in the present PEH is not fast enough to damage the magnet. In addition, the PZT is also brittle and easy to be damaged, although it is not impacted directly by the magnet. A more detail stress analysis could be performed in the future for the damage evaluation of the brittle materials.

## Figures and Tables

**Table 1:** The geometric parameters of the beam and magnets.

Parts	Geometric Parameters
Beam	*L*_0_ = 0.5 mm, *L*_1_ = *L*_2_ = *L*_3_ = *L*_4_ = 10 mm, *L*_5_ = 1.5 mm, *L*_6_ = 5 mm, *t_p_* = 0.239 mm, *t_m_* = 0.1 mm, *b* = 10 mm
Magnet A	*L_a_* = *H_a_* = *W_a_* = 10 mm
Magnet B	*L_b_* = 5 mm, *H_b_* = 1.6 mm, *W_b_* = 10 mm
Magnet C	*L_c_* = *H_c_* = *W_c_* = 10 mm

**Table 2:** The material properties used in the model.

Material	Property
Piezoelectric	*E_pzt_* = 1/*s*_11_ = 66 GPa, *ρ_pzt_* = 7900 kg/m^3^, *d*_31_ = 140 × 10^−12^ C/N, *ε*_33_/*ε*_0_ = 2100
Metal shim	*E_metal_* = 110 GPa, *ρ_metal_* = 8000 kg/m^3^
Magnet	*ρ_mag_* = 7300 kg/m^3^, *M_a_* = 0.9537 T, *M_b_* = 0.5147 T

**Table 3:** Measurement results of *u*_0_, *v_a_*, and impact velocity before impact for the five tests.

Test No.	1	2	3	4	5	Average
*u*_0_ (mm)	7.80	7.79	7.85	7.87	7.75	7.81
*v*_a_ (m/s)	0.113	0.134	0.082	0.080	0.078	0.097
Impact velocity (m/s)	5.451	5.047	5.207	4.372	5.456	5.106

## Appendix A

The normalized deflection $W_{k}^{\left(1\right)}(x_{k})$ appeared in Equation (1) is given by

$$W_{k}^{\left(1\right)}(x_{k})=\{\begin{array}{l} \frac{1}{2{\left(EI\right)}_{0}}\left(\frac{1}{2}L_{6}+\sum_{i=0}^{5}L_{i}\right)x_{0}^{2}-\frac{1}{6{\left(EI\right)}_{0}}x_{0}^{3},\;\mathrm{for}\;k=0\\ \frac{1}{2{\left(EI\right)}_{k}}\left(\frac{1}{2}L_{6}+\sum_{i=k}^{5}L_{i}\right)x_{k}^{2}-\frac{1}{6{\left(EI\right)}_{k}}x_{k}^{3}+\left({\frac{dW_{k-1}^{\left(1\right)}}{dx_{k-1}}|}_{x_{k-1}=L_{k-1}}\right)x_{k}+{W_{k-1}^{\left(1\right)}|}_{x_{k-1}=L_{k-1}},\;\mathrm{for}\;k=1,2,\cdots ,5\\ W_{6}^{\left(1\right)}(x_{6})=\left({\frac{dW_{5}^{\left(1\right)}}{dx_{5}}|}_{x_{5}=L_{5}}\right)x_{6}+{W_{5}^{\left(1\right)}|}_{x_{5}=L_{5}},\;\mathrm{for}\;k=6 \end{array}$$

The interpolation functions appeared in Equation (26) is given by

$$N_{k1}=1-\frac{3x_{k}^{2}}{L_{k}^{2}}+\frac{2x_{k}^{3}}{L_{k}^{3}},\;N_{k2}=x_{k}-\frac{2x_{k}^{2}}{L_{k}}+\frac{x_{k}^{3}}{L_{k}^{2}},\;N_{k3}=\frac{3x_{k}^{2}}{L_{k}^{2}}-\frac{2x_{k}^{3}}{L_{k}^{3}},\;N_{k4}=-\frac{x_{k}^{2}}{L_{k}}+\frac{x_{k}^{3}}{L_{k}^{2}},\;k\;=\;0,\;2,\;\ldots ,\;6$$

The element *M_i_*_-*j*_ (*i*, *j* = 1, 2, …, 11) at *i*th row and *j*th column of the mass matrix **M** appeared in Equation (33) is given by

$$\begin{array}{c} M_{1-1}=\frac{13}{35}[L_{0}{\left(\rho A\right)}_{0}+L_{1}{\left(\rho A\right)}_{1}],\;M_{1-2}=M_{2-1}=-\frac{11}{210}[L_{0}^{2}{\left(\rho A\right)}_{0}-L_{1}^{2}{\left(\rho A\right)}_{1}],\;\\ M_{1-3}=M_{3-1}=\frac{9}{70}L_{1}{\left(\rho A\right)}_{1},\;M_{1-4}=M_{4-1}=-\frac{13}{420}L_{1}^{2}{\left(\rho A\right)}_{1},\;\\ M_{2-2}=\frac{1}{105}[L_{0}^{3}{\left(\rho A\right)}_{0}+L_{1}^{3}{\left(\rho A\right)}_{1}],\;M_{2-3}=M_{3-2}=\frac{13}{420}L_{1}^{2}{\left(\rho A\right)}_{1},\;\\ M_{2-4}=M_{4-2}=-\frac{1}{140}L_{1}^{3}{\left(\rho A\right)}_{1},\;M_{3-3}=\frac{13}{35}[L_{1}{\left(\rho A\right)}_{1}+L_{2}{\left(\rho A\right)}_{2}],\;\\ M_{3-4}=M_{4-3}=-\frac{11}{210}[L_{1}^{2}{\left(\rho A\right)}_{1}-L_{2}^{2}{\left(\rho A\right)}_{2}],\;M_{3-5}=M_{5-3}=\frac{9}{70}L_{2}{\left(\rho A\right)}_{2},\;\\ M_{3-6}=M_{6-3}=-\frac{13}{420}L_{2}^{3}{\left(\rho A\right)}_{2},\;M_{4-4}=\frac{1}{105}[L_{1}^{3}{\left(\rho A\right)}_{1}+L_{2}^{3}{\left(\rho A\right)}_{2}],\;\\ M_{4-5}=M_{5-4}=\frac{13}{420}L_{2}^{2}{\left(\rho A\right)}_{2},\;M_{4-6}=M_{6-4}=-\frac{1}{140}L_{2}^{3}{\left(\rho A\right)}_{2},\;\\ M_{5-5}=\frac{13}{35}[L_{2}{\left(\rho A\right)}_{2}+L_{3}{\left(\rho A\right)}_{3}],\;M_{5-6}=M_{6-5}=-\frac{11}{210}[L_{2}^{2}{\left(\rho A\right)}_{2}-L_{3}^{2}{\left(\rho A\right)}_{3}],\;\\ M_{5-7}=M_{7-5}=\frac{9}{70}L_{3}{\left(\rho A\right)}_{3},\;M_{5-8}=M_{8-5}=-\frac{13}{420}L_{3}^{2}{\left(\rho A\right)}_{3},\;\\ M_{6-6}=\frac{1}{105}[L_{2}^{2}{\left(\rho A\right)}_{2}+L_{3}^{2}{\left(\rho A\right)}_{3}],\;M_{6-7}=M_{7-6}=\frac{13}{420}L_{3}^{2}{\left(\rho A\right)}_{3},\;\\ M_{6-8}=M_{8-6}=-\frac{1}{140}L_{3}^{2}{\left(\rho A\right)}_{3},\;M_{7-7}=\frac{13}{35}[L_{3}{\left(\rho A\right)}_{3}+L_{4}{\left(\rho A\right)}_{4}],\;\\ M_{7-8}=M_{8-7}=-\frac{11}{210}[L_{3}^{2}{\left(\rho A\right)}_{3}-L_{4}^{2}{\left(\rho A\right)}_{4}],\;M_{7-9}=M_{9-7}=\frac{9}{70}L_{4}{\left(\rho A\right)}_{4},\;\\ M_{7-10}=M_{10-7}=-\frac{13}{420}L_{4}^{2}{\left(\rho A\right)}_{4},\;M_{8-8}=\frac{1}{105}[L_{3}^{3}{\left(\rho A\right)}_{3}+L_{4}^{3}{\left(\rho A\right)}_{4}],\;\\ M_{8-9}=M_{9-8}=\frac{13}{420}L_{4}^{2}{\left(\rho A\right)}_{4},\;M_{8-10}=M_{10-8}=-\frac{1}{140}L_{4}^{3}{\left(\rho A\right)}_{4},\;\\ M_{9-9}=\frac{13}{35}[L_{4}{\left(\rho A\right)}_{4}+L_{5}{\left(\rho A\right)}_{5}],\;M_{9-10}=M_{10-9}=-\frac{11}{210}[L_{4}^{2}{\left(\rho A\right)}_{4}-L_{5}^{2}{\left(\rho A\right)}_{5}],\;\\ M_{9-11}=M_{11-9}=\frac{L_{5}\left(13L_{5}+54L_{6}\right){\left(\rho A\right)}_{5}}{420L_{6}},\;M_{10-10}=\frac{1}{105}[L_{4}^{3}{\left(\rho A\right)}_{4}+L_{5}^{3}{\left(\rho A\right)}_{5}],\;\\ M_{10-11}=M_{11-10}=\frac{L_{5}^{2}\left(3L_{5}+13L_{6}\right){\left(\rho A\right)}_{5}}{420L_{6}},\;\\ M_{11-11}=\frac{1}{105L_{6}^{2}}\left[\left(L_{5}^{3}+11L_{5}^{2}L_{6}+39L_{5}L_{6}^{2}\right){\left(\rho A\right)}_{5}+35L_{6}^{3}{\left(\rho A\right)}_{6}\right] \end{array}$$

The element *K_i_*_-*j*_ (*i*, *j* = 1, 2, …, 11) at *i*th row and *j*th column of the stiffness matrix **K** appeared in Equation (33) is given by

It is noted that the elements not mentioned above for the mass matrix and stiffness matrix are zero.

$$\begin{array}{c} K_{1-1}=12[\frac{{\left(EI\right)}_{0}}{L_{0}^{3}}+\frac{{\left(EI\right)}_{1}}{L_{1}^{3}}],\;K_{1-2}=K_{2-1}=6[-\frac{{\left(EI\right)}_{0}}{L_{0}^{2}}+\frac{{\left(EI\right)}_{1}}{L_{1}^{2}}],\;K_{1-3}=K_{3-1}=-\frac{12{\left(EI\right)}_{1}}{L_{1}^{3}},\;\\ K_{1-4}=K_{4-1}=\frac{6{\left(EI\right)}_{1}}{L_{1}^{2}},\;K_{2-2}=4[\frac{{\left(EI\right)}_{0}}{L_{0}}+\frac{{\left(EI\right)}_{1}}{L_{1}}],\;K_{2-3}=K_{3-2}=-\frac{6{\left(EI\right)}_{1}}{L_{1}^{2}},\;\\ K_{2-4}=K_{4-2}=\frac{2{\left(EI\right)}_{1}}{L_{1}},\;K_{3-3}=12[\frac{{\left(EI\right)}_{1}}{L_{1}^{3}}+\frac{{\left(EI\right)}_{2}}{L_{2}^{3}}],\;K_{3-4}=K_{4-3}=6[-\frac{{\left(EI\right)}_{1}}{L_{1}^{2}}+\frac{{\left(EI\right)}_{2}}{L_{2}^{2}}],\;\\ K_{3-5}=K_{5-3}=-\frac{12{\left(EI\right)}_{2}}{L_{2}^{3}},\;K_{3-6}=K_{6-3}=\frac{6{\left(EI\right)}_{2}}{L_{2}^{2}},\;K_{4-4}=4[\frac{{\left(EI\right)}_{1}}{L_{1}}+\frac{{\left(EI\right)}_{2}}{L_{2}}],\;\\ K_{4-5}=K_{5-4}=-\frac{6{\left(EI\right)}_{2}}{L_{2}^{2}},\;K_{4-6}=K_{6-4}=\frac{2{\left(EI\right)}_{2}}{L_{2}},\;K_{5-5}=12[\frac{{\left(EI\right)}_{2}}{L_{2}^{3}}+\frac{{\left(EI\right)}_{3}}{L_{3}^{3}}],\;\\ K_{5-6}=K_{6-5}=6[-\frac{{\left(EI\right)}_{2}}{L_{2}^{2}}+\frac{{\left(EI\right)}_{3}}{L_{3}^{2}}],\;K_{5-7}=K_{7-5}=-\frac{12{\left(EI\right)}_{3}}{L_{3}^{3}},\;K_{5-8}=K_{8-5}=\frac{6{\left(EI\right)}_{3}}{L_{3}^{2}},\;\\ K_{6-6}=4[\frac{{\left(EI\right)}_{2}}{L_{2}}+\frac{{\left(EI\right)}_{3}}{L_{3}}],\;K_{6-7}=K_{7-6}=-\frac{6{\left(EI\right)}_{3}}{L_{3}^{2}},\;K_{6-8}=K_{8-6}=\frac{2{\left(EI\right)}_{3}}{L_{3}},\;\\ K_{7-7}=12[\frac{{\left(EI\right)}_{3}}{L_{3}^{3}}+\frac{{\left(EI\right)}_{4}}{L_{4}^{3}}],\;K_{7-8}=K_{8-7}=6[-\frac{{\left(EI\right)}_{3}}{L_{3}^{2}}+\frac{{\left(EI\right)}_{4}}{L_{4}^{2}}],\;\\ K_{7-9}=K_{9-7}=-\frac{12{\left(EI\right)}_{4}}{L_{4}^{3}},\;K_{7-10}=K_{10-7}=\frac{6{\left(EI\right)}_{4}}{L_{4}^{2}},\;K_{8-8}=4[\frac{{\left(EI\right)}_{3}}{L_{3}}+\frac{{\left(EI\right)}_{4}}{L_{4}}],\;\\ K_{8-9}=K_{9-8}=-\frac{6{\left(EI\right)}_{4}}{L_{4}^{2}},\;K_{8-10}=K_{10-8}=\frac{2{\left(EI\right)}_{4}}{L_{4}},\;K_{9-9}=12[\frac{{\left(EI\right)}_{4}}{L_{4}^{3}}+\frac{{\left(EI\right)}_{5}}{L_{5}^{3}}],\;\\ K_{9-10}=K_{10-9}=6[-\frac{{\left(EI\right)}_{4}}{L_{4}^{2}}+\frac{{\left(EI\right)}_{5}}{L_{5}^{2}}],\;K_{9-11}=K_{11-9}=-\frac{6\left(L_{5}+2L_{6}\right){\left(EI\right)}_{5}}{L_{5}^{3}L_{6}},\;\\ K_{10-10}=4[\frac{{\left(EI\right)}_{4}}{L_{4}}+\frac{{\left(EI\right)}_{5}}{L_{5}}],\;K_{10-11}=K_{11-10}=-\frac{2\left(L_{5}+3L_{6}\right){\left(EI\right)}_{5}}{L_{5}^{2}L_{6}},\;\\ K_{11-11}=k_{eq}+\frac{4\left(L_{5}^{2}+3L_{5}L_{6}+3L_{6}^{2}\right){\left(EI\right)}_{5}}{L_{5}^{2}L_{6}^{2}} \end{array}$$

The electromechanical coupling matrix **α** is given by

$$\alpha =\frac{bd_{31}\left(t_{m}+t_{p}\right)}{s_{11}}{\left[\begin{array}{ccccccccccc} 0 & 1 & 0 & 0 & 0 & 0 & 0 & 0 & 0 & -1 & 0 \end{array}\right]}^{T}$$
